# Addition of a Single Low Dose of Anti T-Lymphocyte Globulin to Post-Transplant Cyclophosphamide after Allogeneic Hematopoietic Stem Cell Transplant: A Pilot Study

**DOI:** 10.3390/jcm11041106

**Published:** 2022-02-19

**Authors:** Elisabetta Xue, Francesca Lorentino, Maria Teresa Lupo Stanghellini, Fabio Giglio, Simona Piemontese, Daniela Teresa Clerici, Francesca Farina, Sara Mastaglio, Alessandro Bruno, Edoardo Campodonico, Rosamaria Nitti, Magda Marcatti, Andrea Assanelli, Consuelo Corti, Fabio Ciceri, Jacopo Peccatori, Raffaella Greco

**Affiliations:** 1 Haematology and Bone Marrow Transplant Unit, IRCCS San Raffaele Scientific Institute, 20132 Milan, Italy; xue.elisabetta@hsr.it (E.X.); lorentino.francesca@hsr.it (F.L.); lupostanghellini.mariateresa@hsr.it (M.T.L.S.); giglio.fabio@hsr.it (F.G.); piemontese.simona@hsr.it (S.P.); clerici.daniela@hsr.it (D.T.C.); farina.francesca@hsr.it (F.F.); mastaglio.sara@hsr.it (S.M.); bruno.alessandro@hsr.it (A.B.); campodonico.edoardo@hsr.it (E.C.); nitti.rosamaria@hsr.it (R.N.); marcatti.magda@hsr.it (M.M.); assanelli.andrea@hsr.it (A.A.); corti.consuelo@hsr.it (C.C.); ciceri.fabio@hsr.it (F.C.); 2 PhD Program in Public Health, Department of Medicine and Surgery, University of Milano-Bicocca, 20126 Milan, Italy

**Keywords:** GvHD prophylaxis, post-transplant cyclophosphamide, anti-T lymphocyte globulin

## Abstract

Correlation between risk of graft-versus-host disease (GvHD) and CD3^+^ counts within the peripheral blood stem cell graft has recently been reported in the setting of post-transplant cyclophosphamide (PT-Cy). We aimed to investigate the benefit of the addition of a single dose of anti-T lymphocyte globulin (ATLG 5 mg/kg) to PT-Cy in this setting. Starting in 2019, all patients receiving PBSC transplant containing CD3^+^ counts above 300 × 10^6^/kg (study group) received a post-transplant dose of ATLG in addition to standard PT-Cy. The study was designed as a real-life analysis and included all consecutive Hematopoietic Stem Cell Transplantation (HSCT) recipients according to the above-mentioned inclusion criterion (*n* = 21), excluding cord blood and bone marrow donors. Using a 1:2 matched-pair analysis, we compared the outcomes with a historical population who received PT-Cy only (control group). We found a delayed platelet engraftment (29% vs. 45% at 30 days, *p* = 0.03) and a non-significant trend toward higher risk of poor graft function (29% vs. 19%, *p* = 0.52). The addition of ATLG impacted long-term immune reconstitution on the CD4^+^ subsets, but this did not translate into higher rate of relapse or viral infection. Acute GvHD was not significantly impacted, but 1-year cumulative incidence of chronic GvHD was significantly lower in the study group (15% vs. 41%, *p* = 0.04). Survival outcomes were comparable. In conclusion PT-Cy and ATLG was overall safe and translated into a low rate of chronic GvHD incidence.

## 1. Introduction

Despite the recent advances in prophylaxis and management, graft-versus-host disease (GvHD) still represents a major challenge in allogeneic hematopoietic cell transplants (HSCT), with significant impact on long-term quality of life [1]. The association of calcineurin inhibitors (CNI) with antimetabolites has been the backbone of GvHD prophylaxis; however, alternative strategies have been developed. The administration of anti-T-lymphocyte globulin (ATLG), as an in vivo T cell depletion strategy, has been shown to significantly decrease the chronic GvHD (cGvHD) rate in both matched related (MRD) and unrelated (MUD) donor settings, although the benefit on survival outcomes is still a matter of debate [2,3,4]. More recently, the use of post-transplant cyclophosphamide (PT-Cy), initially introduced in haploidentical transplants, has been investigated in other HSCT settings, showing comparable outcomes with ATLG-containing platforms [5,6,7]. Preliminary data are available on the combination of pre-transplant thymoglobulin with PT-Cy, with heterogeneous results [8,9,10,11,12]. 

The growing use of peripheral blood stem cell (PBSC) grafts and HLA-mismatched HSCT donors has been associated with increasing rates of cGvHD [13].

Graft composition also plays a role, with higher CD3^+^ counts within the graft being associated with increased risk of both aGvHD [14] and cGvHD [15] and negatively impacting survival outcomes in adult patients receiving both HLA-mismatched and HLA-matched PBSC transplants. 

In our study, we aimed to evaluate safety and efficacy in preventing GvHD of the post-transplant co-administration of low dose ATLG with cyclophosphamide in a CNI-free population receiving a high CD3^+^ content in the transplanted graft. 

## 2. Patients and Methods

In our institution, all patients transplanted for a hematologic disorder who received PBSC graft containing a CD3^+^ count above 300 × 10^6^/kg were routinely given PT-Cy on day 3 and day 4 at the daily dose of 50 mg/kg, as per standard practice, plus one single administration of ATLG (Grafalon, Neovii) on day 5 at the dose of 5 mg/kg, with the aim of minimizing GvHD risk. Both matched and mismatched and related and unrelated donors were included. All hematologic malignancies and all disease status were included. 

Conditioning regimen was based on treosulfan and fludarabine, with or without the addition of second alkylate agent (melphalan or thiotepa) or total body irradiation [16,17]. Sirolimus administration started on day 5 and was modulated according to therapeutic drug monitoring (TDM); patients transplanted from HLA-mismatched or unrelated donors received also mycophenolic acid (MMF) at the daily dose of 30 mg/kg from day 5 to day 28 [18]. According to center clinical practice, we adopted a different desired target for transplanted CD34^+^ of 5–6 × 10^6^/kg until late 2018, and of 7 × 10^6^/kg after 2019. 

Antimicrobial prophylaxis was based on azoles, cotrimoxazole, and acyclovir as per institutional guidelines; 15 patients also received letermovir prophylaxis. Monitoring of cytomegalovirus (CMV), herpes six virus (HHV6), adenovirus (AdV), and Epstein–Barr virus (EBV) viremia and aspergillus antigen were performed weekly until day 100; afterwards, CMV and EBV viremia were monitored every other week until immune suppressants discontinuation. Starting in May 2020, we added the routine prophylactic use of granulocyte colony-stimulating factors (G-CSF) in all transplant recipients, given as a single peg-filgrastim dose or daily filgrastim until engraftment. This change in clinical practice was introduced due to the ongoing COVID-19 pandemic, with the aim of shortening the neutropenic phase and patients’ in-hospital stay. 

Neutrophil engraftment was defined as achievement of an absolute polymorphonuclear leukocyte count above 500 cells/μL for 3 consecutive days. Poor graft function was defined as a bi- or tri-lineage cytopenia after day 28 in the presence of full donor hypoplastic marrow and of disease remission [19]. We collected data on chimerism data measured through real-time quantitative polymerase chain reaction on bone marrow samples performed around day 30 for patients with disease remission [20]. Acute GvHD was scored following the CIBMTR Severity Index [21,22], while chronic GvHD was scored according to the National Institutes of Health consensus criteria [23]. Transplant-related mortality (TRM) was defined as death from any cause while in continuous remission of the primary disease. Overall survival (OS) was defined as the interval from HSCT to death, and patients were censored at the date of last contact if alive. Progression-free survival (PFS) was defined as the interval from HSCT to either relapse/progression or death in remission, whichever came first.

We compared the results from this population with a historical cohort of patients transplanted from 2015 to 2018 with a PBSC graft containing CD3^+^ counts above 300 × 10^6^/kg who received PT-Cy, sirolimus (with or without MMF, as described above), without additional ATLG. To avoid confounding factors, each patient receiving ATLG (case) was matched with two patients not receiving ATLG (control) for the following criteria: hematological disease type and status at HSCT, patient–donor HLA matching (i.e., matched related, mismatched related (i.e., haploidentical), matched unrelated, mismatched unrelated), donor’s age, conditioning intensity, and CD3^+^ count within the graft. Matching was performed on the logit of propensity score [24].

All outcomes were measured from the time of stem cell infusion. Patient-, disease-, and transplant-related characteristics were compared using the χ^2^ or Fisher’s exact test for categorical variables and the Mann–Whitney U-test for continuous variables. 

Probabilities of OS and PFS were calculated using the Kaplan–Meier semi-parametric estimator, and groups were compared using the log-rank test [25,26]. 

Cumulative incidence functions were used to estimate engraftment, GvHD, relapse, and TRM. Relapse and death for any cause was a competing event for engraftment and GvHD. Relapse was a competing event for TRM and vice versa. Gray’s test was used for comparisons of cumulative incidence functions [27,28]. 

Analyses were performed using R version 4.0.4 (http://www.R-project.org (accessed on 6 November 2021); propensity score matching was performed using the MatchIt package (https://cran.r-project.org/web/packages/MatchIt/MatchIt.pdf (accessed on 6 November 2021).

## 3. Results

We identified twenty patients transplanted from January 2019 to August 2021 who received the combination of PT-Cy plus low dose ATLG (ATLG; Grafalon, Neovii Biotech, Lexington, MA) as the GvHD prophylaxis (study group); data from one single patient transplanted in 2015 who respected the same inclusion criterion were included in the analysis. Both first and second transplants were included. Within the ATLG-free cohort (control group), we identified 42 patients transplanted between June 2015 and December 2018 with whom to perform the matched-paired analysis. Patient and transplant characteristics are shown in Table 1. A total of 10 out of 21 patients (48%) of the study group received prophylactic G-CSF, vs. none in the control group.

The study group follow up was shorter, with a median of 538 days (interquartile range, IQR 439–664 days) vs. 1450 days (IQR 1188–1634 days) in the control group, due to different transplant timeframe. 

In the study group, median counts of CD34^+^ and CD3^+^ in the transplanted grafts were 5.88 × 10^6^/kg (range 3.06–7.73) and 464 × 10^6^/kg (range 254–672), respectively, whereas in the control group, median counts of CD34^+^ and CD3^+^ in the transplanted grafts were 6.99 × 10^6^/kg (range 2.88–10.87) and 399 × 10^6^/kg (range 347–511), respectively.

In the study group, neutrophil engraftment occurred in 19 patients after a median of 19 days (range 14–37), and platelet engraftment occurred in 13 patients after a median of 34 days post-HSCT (range 15–68); 6 patients experienced poor graft functions (PGF), 3 of whom required CD34^+^-selected boost to restore hematopoiesis. Two patients died in aplasia before engraftment could occur. None had graft rejection. No differences in time to engraftment and rate of PGF were found between HLA-matched and HLA-mismatched transplants. 

When compared with the control group, we found no difference in 30-day cumulative incidence of neutrophil engraftment (81%, 95% confidential interval, CI 59–93 in the study group vs. 69%, 95% CI 52–81 in the control group, *p* = 0.19). Rather, cumulative incidence of platelet engraftment was significantly lower in the study group at both 30 days (29%, 95% CI 11–49 vs. 45%, 95% CI 30–60 in the control group, *p* = 0.03) and at 60 days post-HSCT (52%, 95% CI 29–72 vs. 71%, 95% CI 55–83 in the control group, *p* = 0.03). At day 30, there was no difference in chimerism between the two groups (median of 0.3% host and 0.7% host in the study and in the control group, respectively, *p* = 0.1). In the control group, eight patients experienced PGF, and two required CD34^+^-selected boost. One patient died in aplasia due to disease persistence. There was a non-significant trend for 100-day higher cumulative incidence of PGF, which was 29% (95% CI 11–49) in the study group vs. 19% (95% CI 9–32) in the control group, *p* = 0.52. There was no significant difference in terms of patient–donor ABO compatibility between the two groups (8/21 vs. 23/42, *p* = 0.2).

The 100-day cumulative incidence of CMV clinically relevant reactivation was 14% (95% CI 3–33) in the study group and 31% (95% CI 18–45) in the control group (*p* = 0.33). However, fifteen patients from the study group received letermovir prophylaxis vs. none in the control group; thus, no conclusions can be drawn with regards to the ATLG effect on CMV activation. In the study group, 12 patients had HHV6 reactivation, including 2 CNS localizations and 3 gastrointestinal tract localizations, for a 100-day cumulative incidence of 57% (95% CI 33–76) vs. 50% (95% CI 34–64) in the control group (*p* = 0.46). All but one HHV6 reactivation occurred before day 100. We found no differences in AdV reactivation (1/21 in study group vs. 5/42 control group, *p* = 0.65), EBV reactivation (3/21 in study group vs. 5/42 control group, *p* = NS), and BK urinary tract infection (5/21 in study group vs. 9/42 in control group, *p* = NS). We documented only one case of post-transplant lymphoproliferative disorder, which occurred in the control group. 

In patients receiving ATLG, we documented a high incidence of invasive fungal infection (IFI), which occurred in 7 out of 21 cases after a median of 80 days post-HSCT (range 11–216 days) and consisted in 4 probable and 1 possible aspergillosis, 1 invasive candidiasis, and 1 disseminated Scedosporium spp. infection [29]. IFI rate was lower in the control group (9/42 cases, consisting in 5 probable and 2 possible aspergilloses, 2 candidiasis), although this difference was non-significant (*p* = 0.36).

**Figure 1:**
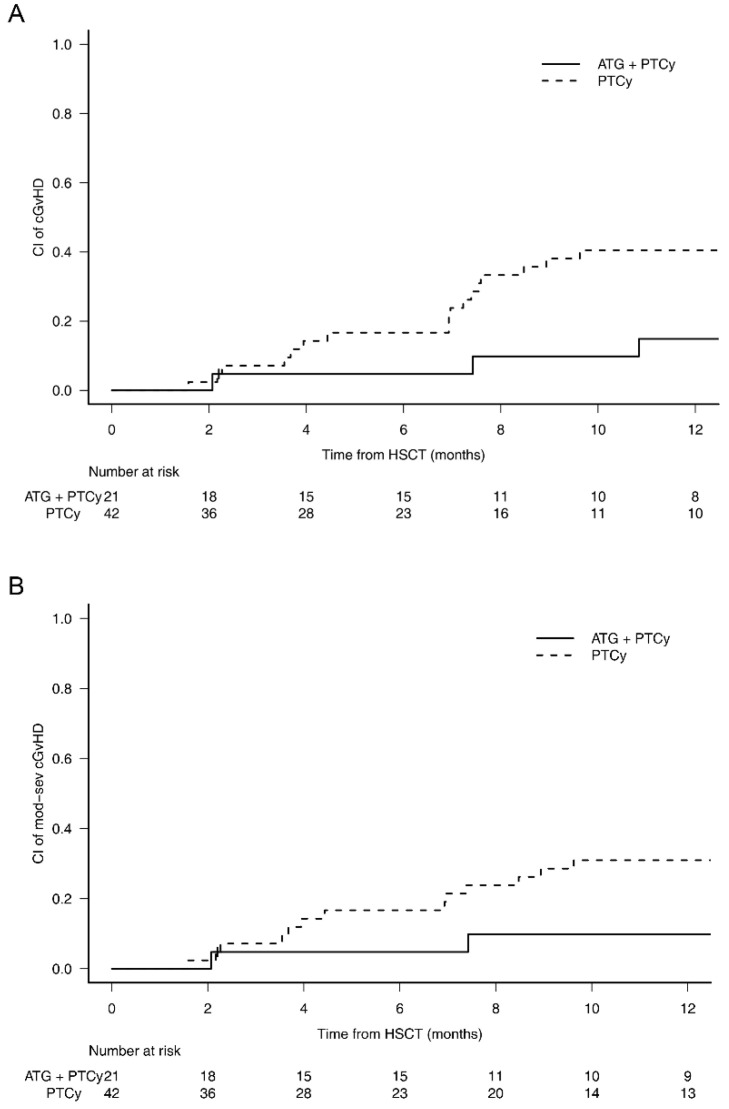
Chronic GvHD. Patients with a follow-up longer than 100 days were evaluated for cGvHD. (**A**) In the study group, 1-year cumulative incidence of cGvHD was 15% (95% CI 3–34) vs. 41% (95% CI 25–55) in the control group, *p* = 0.04. (**B**) In the study group, 1-year cumulative incidence of moderate-to-severe cGvHD was 10% (95% CI 2–28) vs. 31% (95% CI 18–45) in the control group, *p* = 0.07. Abbreviations: PT-Cy, post-transplant cyclophosphamide; ATG anti-T lymphocyte globulin; GvHD, Graft versus Host Disease; HSCT, hematopoietic stem cell transplant.

Median time of immune suppressants discontinuation was 199 days (range 129–483 days) and 205 days (range 81–1123 days) in the study group and the control group, respectively. Acute GvHD rates were comparable, with a 100-day cumulative incidence of grade 2–4 aGvHD of 24% (95% CI 8–44) in the study group vs. 29% (95% CI 16–43, *p* = 0.86) in the control group, and of grade 3–4 aGvHD of 10% (95% CI 2–27) vs. 19% (95% CI 9–32, *p* = 0.48), respectively. Patients with a follow-up longer than 100 days were evaluated for cGvHD. Three patients of the study group developed cGvHD after a median of 226 days (range 63–330), including one mild and two moderate forms, whereas eighteen patients from the control group developed cGvHD after a median of 212 days (range 48–707), including four mild, eight moderate, and six severe forms. In the study group, cGVHD involved skin (*n* = 2), liver (*n* = 2), eye (*n* = 1), and mouth (*n* = 1); in the control group, it involved skin (*n* = 10), liver (*n* = 2), eye (*n* = 12), mouth (*n* = 7), fascia/joints (*n* = 2), gastrointestinal tract (*n* = 2), and lung (*n* = 1). ATLG was associated with a significant reduction in 1-year cumulative incidence of cGvHD: 15% (95% CI 3–34) in the study group vs. 41% (95% CI 25–55) in the control group, *p* = 0.04. A trend towards reduced cGvHD was still noticeable when focusing only on moderate-to-severe cGvHD, with 10% (95% CI 2–28) in the study group vs. 31% (95% CI 18–45) in the control group, *p* = 0.07 (Figure 1). 

Immune reconstitution data are shown in Table 2. In the study group, patients achieved CD3^+^ counts above 100 cells/μL a median of 60 days after HSCT (range 25–293), vs. 35 days (range 21–104) in patients who did not receive ATLG. At one month, CD3^+^, CD4^+^, and CD8^+^ counts were significantly lower in patients who received ATLG; over time, the negative impact of ATLG was predominant and long lasting only on the CD4^+^ subsets (*p* = 0.008).

At the last follow-up, 52% (11/21) and 40% (17/42) patients were alive and in disease remission in the study and control group, respectively. Survival outcomes were comparable between the study group and the control group: 1-year TRM was 19% (95% CI 6–39) vs. 19% (95% CI 9–32, *p* = 0.9); 1-year relapse rate was 25% (95% CI 9–46) vs. 24% (95% CI 12–38, *p* = 0.9); 1-year PFS was 56% (95% CI 32–74) vs. 57% (95% CI 41–70, *p* = 0.9), 1-year OS was 75% (95% CI 50–89) vs. 69% (95% CI 53–81, *p* = 0.49). 

## 4. Discussion

Despite advances in optimization of conditioning regimen and GvHD prophylaxis platform, GvHD is still an unmet issue with significant impact on transplant outcomes. Efforts are being made to better identify patients at higher risk and to move toward a patient-tailored GvHD prophylaxis. Mussetti and colleagues previously reported the correlation between high CD3^+^ counts within the graft and higher incidence of cGvHD [15].

In our study, we evaluated the safety and efficacy of the combination of standard PT-Cy dose with low dose of post-HSCT ATLG in patients who received a CD3^+^ content above 300 × 10^6^/kg within the PBSC-transplanted graft; the indication was given irrespective of donor type or hematologic disease. ATLG was administered after transplant with the intent to avoid a reduction in PT-Cy efficacy.

ATLG was overall well tolerated. We found a slower platelet engraftment in patients who received combination of PT-Cy and ATLG, whereas neutrophil engraftment had comparable rates. This observation could be explained by the higher use of prophylactic G-CSF in the study group, which might have skewed hemopoiesis toward granulocyte differentiation. Although we reported a high rate of poor graft function, we did not observe any statistically significant difference between the two groups. We speculate that PT-Cy adoption in both groups, which has a known impact on viral reactivation rates, might have a role in partially explaining the PGF occurrence in both groups, regardless of graft characteristics. However, we also cannot exclude an indirect impact of post-transplant ATLG in delaying hematopoietic engraftment, as a consequence of impact on lymphocytes abrogation.

As expected, indeed, T-cell immune reconstitution was negatively impacted by ATLG use, and the effect on CD4^+^ subsets was evident even one year after transplant. Interestingly, this did not translate into a higher relapse incidence and higher viral infection rate. Unfortunately, no data on virus-specific immunity after HSCT are available, warranting further studies on this topic. Whereas the non-homogeneous anti-CMV prophylaxis between the two groups prevent drawing any conclusions in terms of ATLG’s effect on CMV reactivation, HSV6, AdV, BK, and EBV also showed comparable reactivation rates. Moreover, the potential effect on long-term HSCT outcomes by letermovir prophylaxis might need further investigation. Importantly, ATLG was not associated with higher rate of EBV-related PTLD. The use of PT-Cy has already been associated with high early post-transplant viral infection in several HSCT settings [30,31,32]; however, the co-administration of a low dose of ATLG does not seem to further increase this risk. Although larger studies focusing also on late-onset infections and vaccine responses in the long-term follow-up are needed, we hypothesize that a decreased time on immunosuppressant due to a lower cGvHD rate might reduce viral infection rate, thus balancing the impaired T cell reconstitution in ATLG-recipients. 

Nevertheless, there was a slight, non-significant increase in IFI rate, pointing at the need for strict monitoring in these patients. Indeed, although azole prophylaxis has also remarkably reduced the risk of IFI in the high-risk population [33,34], in more recent years we observed a higher incidence of breakthrough IFI during azole administration [29,35].

Our GvHD rate in the control group falls in line with previous studies using PT-Cy and PBSC [36,37]. We documented a significant reduction in chronic GvHD overall, whereas the impact on moderate–severe cGvHD and on acute GvHD was less evident, and there was a slight, non-significant decrease in grade 3–4 aGvHD in the ATLG-group. Larger studies are needed to determine whether ATLG combined with PT-Cy has impacted the organs involved in cGvHD. We could not find any difference in terms of overall survival between the groups, which is likely due to the constant improvements in management of cGvHD and of long-term complications; thus, we believe that the impact of improving cGvHD prophylaxis would not reflect on better survival, but on better quality of life [38].

Our study has several limitations, starting from its retrospective nature and small population; furthermore, despite matching for main patients’ disease and transplant characteristics, the two groups showed some inhomogeneity: control group patients were transplanted in a prior timeframe and had a longer follow up, whereas study group patients had more use of letermovir and of G-CSF, which might have influenced some of the outcomes. 

Optimal dose and timing of ATLG, as well as patients’ selection for GvHD intensification is unknown. Combination of PT-Cy and ATLG/ATG has been previously reported in both matched and mismatched transplants, with heterogeneous results; in most cases authors adopted thymoglobulin, which was administered pre-transplant. In these trials, different doses of both pre-transplant thymoglobulin and PT-Cy have been described, including single PT-Cy dose. Most [8,9,10,11,12,39,40,41] but not all studies [42] observed a decrease in acute and chronic GvHD rate. Wang and colleagues investigated the addition of low PT-Cy dose to standard thymoglobulin; the authors suggested that low-dose PT-Cy could facilitate suppressive T regs reconstitution and could promote the protective effect of ATG on GvHD [11,43]. Khanolkar and colleagues recently reported the outcomes of a phase II study investigating intensification of GvHD prophylaxis with ATG based on IL-2 levels on day + 7; the authors observed an effective reduction in GvHD rate, at the expense of higher infection-related mortality [44]. The high infection rate reported in several of these studies might be partially explained by either the high dose of thymoglobulin used or by the post-transplant administration. 

In conclusion, early identification of patients at higher risk of developing high grade GvHD, who thus might benefit from an intensification of GvHD prophylaxis, is still to be defined. In our study, we adopted as the criterion the CD3^+^ graft content, based on previous studies that pointed out a correlation between CD3^+^ graft content and GvHD [15,45]. Studies from the last decade have focused on gut microbiota [46] and biomarkers [47,48] as well as machine learning algorithms [49] to predict GvHD occurrence. Moreover, the best dose and schedule of GvHD prophylaxis, including the combination ATLG and PT-Cy, is still to be defined. We speculate that if these and future studies would allow better stratification of the risk, intensification of GvHD prophylaxis, such as combination of ATLG with PT-Cy, might be investigated in the high-risk population. 

## Figures and Tables

**Table 1:** Patient and transplant characteristics.

	Study Group PT-Cy + ATLG N = 21	Control Group PT-Cy N = 42	*p* Value
Median patient age at HSCT (range)	60 (24–71)	56 (22–77)	0.691
Median donor age at HSCT (range)	41 (18–68)	34 (18–70)	0.41
Gender			
Female	N = 9	N = 16	0.71
Male	N = 12	N = 26	
Disease type			
ALL	N = 2	N = 6	0.974
AML	N = 13	N = 23	
MPN/MDS MM/Lymphoma	N = 4 N = 2	N = 8 N = 5	
Disease status at HSCT			
CR1	N = 7	N = 13	1.0
CR>1	N = 7	N = 13	
Not in CR	N = 7	N = 16	
HCT-CI score			
0–1	N = 11	N = 21	0.85
≥2	N = 10	N = 21	
Type of HSCT			
MRD	N = 4	N = 7	0.881
MUD	N = 4	N = 12	
MMUD	N = 4	N = 7	
MMRD	N = 9	N = 16	
Number of allo-HSCT			
First	N = 19	N = 38	1.0
Second	N = 2	N = 4	
Conditioning regimen ^$^			
MAC	N = 13	N = 30	0.567
RTC	N = 8	N = 12	
Median CD3^+^-infused (10^6^/kg, IQR)	464 (409–496)	399 (347–511)	0.197

^$^ Myeloablative regimen included treosulfan–fludarabine–melphalan, treosulfan–fludarabine–thiotepa, treosulfan–fludarabine–TBI; reduced toxicity regimen included treosulfan–fludarabine. Abbreviations: PT-Cy, post-transplant cyclophosphamide; ATLG, anti T lymphocyte globulin; HSCT, hematopoietic stem cell transplant; CR, complete remission; MRD, matched related donor; MUD, matched unrelated donor; MMUD, mismatched unrelated donor; MMRD, mismatched related donor; MAC, myeloablative; RTC, reduced toxicity conditioning; IQR, interquartile range.

**Table 2:** T cell immune reconstitution.

	Study Group PT-Cy + ATLG *n* = 21	Control Group PT-Cy *n* = 42	*p* Value
CD3^+^, median (IQR)			
D + 30	47 (16–91)	104 (52–315)	**0.00783**
D + 90	344 (98–781)	653 (450–982)	0.0866
D + 180	1010 (653–1266)	1012 (716–1852)	0.477
D + 365	1172 (762–1827)	1682 (1063–2423)	0.188
CD4^+^, median (IQR)			
D + 30	14 (6–35)	42 (24–121)	**0.00179**
D + 90	106 (50–222)	206 (164–291)	0.0683
D + 180	176 (142–346)	315 (207–491)	**0.0104**
D + 365	233 (194–402)	539 (352–666)	**0.00842**
CD8^+^, median (IQR)			
D + 30	16 (8–50)	55 (16–144)	**0.0269**
D + 90	217 (54–553)	375 (240–696)	0.118
D + 180	715 (445–1008)	736 (355–1339)	0.75
D + 365	912 (501–1472)	1069 (690–1737)	0.473

Abbreviations: PT-Cy, post-transplant cyclophosphamide; ATLG, anti T lymphocyte globulin; IQR, interquartile range.

## Data Availability

The datasets generated for this study are available on request to the corresponding authors.
